# Early Back-to-Africa Migration into the Horn of Africa

**DOI:** 10.1371/journal.pgen.1004393

**Published:** 2014-06-12

**Authors:** Jason A. Hodgson, Connie J. Mulligan, Ali Al-Meeri, Ryan L. Raaum

**Affiliations:** 1 Department of Life Sciences, Silwood Park Campus, Imperial College London, Ascot, Berkshire, United Kingdom; 2 Department of Anthropology and the Genetics Institute, University of Florida, Gainesville, Florida, United States of America; 3 Department of Biochemistry and Molecular Biology, Sana'a University, Sana'a, Yemen; 4 Department of Anthropology, Lehman College and The Graduate Center, The City University of New York, Bronx, New York, New York, United States of America; 5 The New York Consortium in Evolutionary Primatology (NYCEP), New York, New York, United States of America; Dartmouth College, United States of America

## Abstract

Genetic studies have identified substantial non-African admixture in the Horn of Africa (HOA). In the most recent genomic studies, this non-African ancestry has been attributed to admixture with Middle Eastern populations during the last few thousand years. However, mitochondrial and Y chromosome data are suggestive of earlier episodes of admixture. To investigate this further, we generated new genome-wide SNP data for a Yemeni population sample and merged these new data with published genome-wide genetic data from the HOA and a broad selection of surrounding populations. We used multidimensional scaling and ADMIXTURE methods in an exploratory data analysis to develop hypotheses on admixture and population structure in HOA populations. These analyses suggested that there might be distinct, differentiated African and non-African ancestries in the HOA. After partitioning the SNP data into African and non-African origin chromosome segments, we found support for a distinct African (Ethiopic) ancestry and a distinct non-African (Ethio-Somali) ancestry in HOA populations. The African Ethiopic ancestry is tightly restricted to HOA populations and likely represents an autochthonous HOA population. The non-African ancestry in the HOA, which is primarily attributed to a novel Ethio-Somali inferred ancestry component, is significantly differentiated from all neighboring non-African ancestries in North Africa, the Levant, and Arabia. The Ethio-Somali ancestry is found in all admixed HOA ethnic groups, shows little inter-individual variance within these ethnic groups, is estimated to have diverged from all other non-African ancestries by at least 23 ka, and does not carry the unique Arabian lactase persistence allele that arose about 4 ka. Taking into account published mitochondrial, Y chromosome, paleoclimate, and archaeological data, we find that the time of the Ethio-Somali back-to-Africa migration is most likely pre-agricultural.

## Author Summary

The Horn of Africa (HOA) occupies a central place in our understanding of modern human origins. This region is the location of the earliest known modern human fossils, a possible source for the out-of-Africa migration, and one of the most genetically and linguistically diverse regions of the world. Numerous genetic studies over the last decades have identified substantial non-African ancestry in populations in this region. Because there is archaeological, historical, and linguistic evidence for contact with non-African populations beginning about 3,000 years ago, it has often been assumed that the non-African ancestry in HOA populations dates to this time. In this work, we find that the genetic composition of non-African ancestry in the HOA is distinct from the genetic composition of current populations in North Africa and the Middle East. With these data, we demonstrate that most non-African ancestry in the HOA cannot be the result of admixture within the last few thousand years, and that the majority of admixture probably occurred prior to the advent of agriculture. These results contribute to a growing body of work showing that prehistoric hunter-gatherer populations were much more dynamic than usually assumed.

## Introduction

The timing and extent of migration and admixture are questions that are central to the entire scope of human evolutionary history from the origin of our species to the present day. The most important event underlying human population structure is the origin of anatomically modern humans in Africa and their subsequent migration around the globe [1]–[3]. Following the initial out-of-Africa migration, the rate of migration between sub-Saharan Africa and the rest of the Old World was low throughout prehistory, but not absent; there is statistically significant evidence for a deep history of intercontinental migration [4]–[7]. Beginning around 11 ka (thousand years ago), the switch to reliance on domesticated plants and animals is associated with major population and language expansions from multiple centers of domestication around the world [8]–[10]. Finally, migration and admixture accelerated during the last few thousand years with increasing international trade, including the trade in slaves and the transplantation and shuffling of populations in the colonial era, culminating in the modern era of high international migration.

Populations in the Horn of Africa (HOA: Ethiopia, Eritrea, Djibouti, and Somalia) have substantial non-African ancestry [11]–[15]. The most recent genomic studies estimate 30–50% non-African ancestry in the Cushitic and Semitic speaking populations of the HOA resulting primarily from admixture around 3 ka [16], [17]. This timeframe corresponds to the estimated time of origin of the Ethiosemitic languages [18] and there are some carved inscriptions in South Arabian scripts associated with temple ruins and ritual items in South Arabian styles dated to the early first millennium BCE in the north Ethiopian highlands [19]–[23]. These linguistic and archaeological connections have been cited in the recent population genomic studies to support a hypothesis of high levels of non-African migration into the HOA around 3 ka.

However, more recent archaeological research shows that non-African influences in the HOA were limited and transient. Of the early first millennium BCE inscriptions in non-African scripts complete enough to identify a language, only a small proportion are written in a non-African (South Arabian) language - the majority are written in indigenous proto-Ge'ez [24]. In the HOA, architecture with non-African (primarily South Arabian) elements is entirely monumental or ritual [25] and ritual items with exclusively non-African elements are rare [26]. There are few to no indications of non-African material culture in everyday objects: the ceramics and lithics found outside of the ritual context are almost entirely indigenous with clear local precedents [24], [25], [27]. While earlier scholarship conceived of a South Arabian origin D'MT polity with sovereignty over much of the northern HOA, it is now clear that this polity, if it ever existed at all as an integrated state [24], was geographically restricted to the regions around Yeha and Aksum in what is now the Tigray region of Ethiopia [25]. Artifacts with non-African features are effectively absent in the material culture (ritual or otherwise) of contemporaneous populations in the Eritrean highlands on the Asmara plateau (the “Ancient Ona”) [25], [28], [29]. Prior to the first millennium BC, the archaeology of the HOA is less well studied, but what is available shows no substantial non-African material culture beyond trade relations [25]. Taken all together, the archaeological data could be consistent with limited non-African (primarily South Arabian) migration into the north Ethiopian highlands at the outset of the first millennium BCE, but cannot support large-scale population movements from any foreign population.

Archaeological data indicate trade between the HOA and Arabia by at least 8 ka [30], [31] and genetic analyses of mitochondrial and Y chromosome data suggest much earlier migrations into the HOA. Mitochondrial data are suggestive of as many as three waves of prehistoric non-African migration into the HOA. First, HOA populations carry several unique M1 lineages of the otherwise South and East Asian mitochondrial haplogroup M [13], [32]–[34]. Many of these HOA M1 lineages have deep roots, diverging from M1 representatives elsewhere 20–30 ka [34]–[36]. Second, representatives of N1a and N2a in the HOA diverged from their most closely related haplotypes in the Middle East and the Caucasus 15–20 ka [37]. Third, in the Eurasian mitochondrial HV1 and R0a lineages there are several sub-haplogroups (HV1a3, HV1b1, R0a2b, R0a2g) that are found in both the HOA and the Arabian Peninsula. Within these shared sub-haplogroup lineages, the HOA and Arabian haplotypes are distinct, suggesting that the migration that brought these lineages into the HOA happened soon after the sub-haplogroups began to diversify at 6–10 ka [38], [39].

Y chromosome data are also suggestive of at least two episodes of non-African migration into the HOA prior to 3 ka. First, HOA populations carry E-M78 Y chromosomes at high frequencies [40], [41]. E-M78 originated in northeastern Africa around 19 ka with a descendant lineage (E-V32) unique to the HOA that arrived by at least 6 ka [41]. Because northern African populations in this timeframe are inferred to have substantial non-African ancestry [42], [43], the expansion south of E-M78 could have introduced non-African ancestry into the HOA prior to 6 ka. Second, some HOA populations carry moderate to high frequencies of T-M70 (previously K2-M70) Y chromosomes [44]–[46]. The T haplogroup originated in the area of the Levant approximately 21 ka and the T-M70 sub-haplogroup was present in northeast Africa by at least 14 ka, possibly arriving in the HOA as early as 5 ka [44], [45], [47].

In order to investigate the discrepancy among the archaeological, historical, mitochondrial, Y chromosome, and genome-wide data for recent vs. more ancient evidence of admixture in the HOA, we generated new genome-wide SNP data for a Yemeni sample and analyzed these new data with publicly available data [16], [43], [48]–[51]. Our objectives were to verify the presence of admixture in the HOA, determine the affinities of any HOA non-African ancestry, and evaluate the number of distinct admixture episodes and their timing.

## Results and Discussion

For these analyses, we generated new genome-wide SNP data using the Illumina 370K array from 61 Yemenis, chosen to represent all geographic regions of the country. These new data were merged with published data from the HOA [16], the Middle East [48], North Africa [43], Qatar [50], southern Africa [51], west Africa [49], the HapMap3 project [52], and the Human Genome Diversity Project [53]. After reduction to SNPs shared across all source datasets and quality control, the main merged dataset included 2,194 individuals from 81 populations for 16,766 SNPs (Table S1).

### Horn of Africa populations in the regional genetic landscape

**Figure 1:**
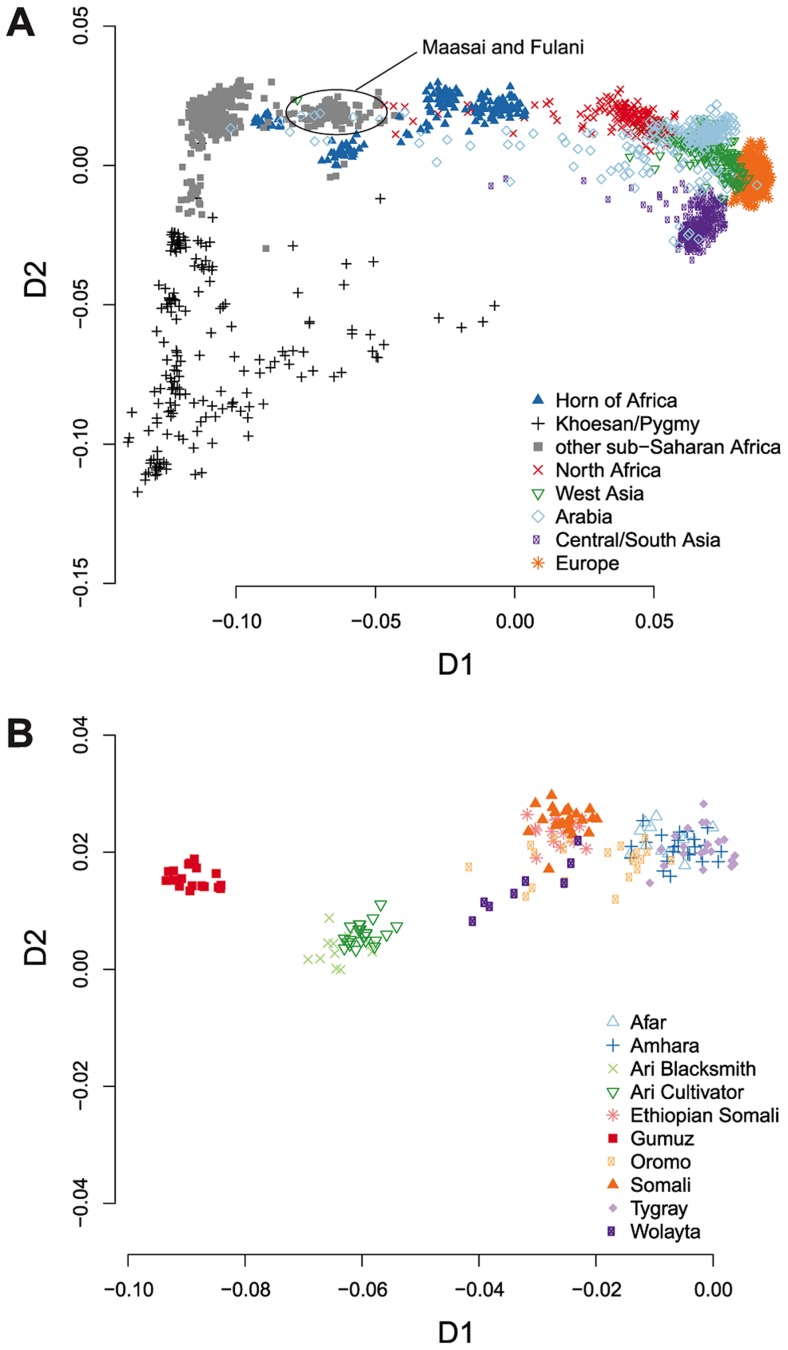
Multidimensional scaling analysis shows the great genetic diversity within the Horn of Africa. We plotted the first two dimensions of a multidimensional scaling analysis of pairwise identity by state across all study populations. (A) The HOA populations are broadly scattered between out-of-African populations and the bulk of sub-Saharan African populations along the first dimension. Some clusters of HOA individuals are much closer to the main sub-Saharan African cluster, while others are much closer to North African and Arabian clusters. (B) In this plot, we zoom in on the HOA samples and leave out all other populations. While the region as a whole covers a broad swath of the first MDS dimension, most individual populations are tightly clumped, with groups separated by language. The Nilo-Saharan speaking Gumuz are on the far left, the Omotic speaking Ari are in the center, and the Cushitic and Semitic speaking populations are on the right.

We first investigated the position and dispersion of HOA populations in the genetic landscape in a multi-dimensional scaling (MDS) analysis of pairwise identity by state (IBS). Consistent with prior analyses of global genome-wide genetic variation [3], [53], [54], the first dimension of the IBS MDS analysis separates sub-Saharan Africans from non-Africans (Figure 1A). The HOA samples are broadly dispersed between the main sub-Saharan Africa cluster and the non-African populations and several sub-clusters of HOA samples are apparent. To see the specific distribution of all of the included HOA samples, we plotted the HOA samples in isolation (Figure 1B). While we include many more African and non-African population samples than prior analysis of these HOA data, our results in the MDS analysis for the HOA samples are not qualitatively different than those of Pagani et al. [16], who showed that the different HOA clusters correspond to linguistic groups: the Gumuz are Nilotic-speaking, the Ari and Wolayta are Omotic-speaking, and the rest speak Cushitic or Semitic languages. The dispersion of HOA samples between the sub-Saharan and non-African clusters is suggestive of admixture between African and non-African ancestors [55], [56].

**Figure 2:**
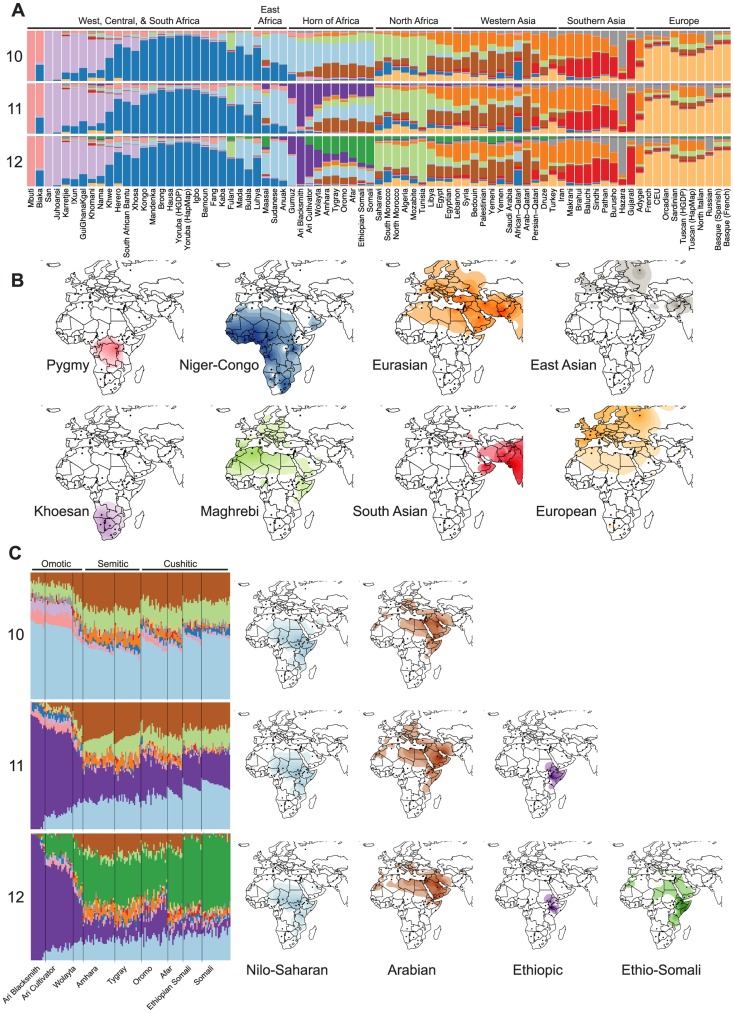
Population structure of Horn of Africa populations in a broad context. ADMIXTURE analysis reveals both well-established and novel ancestry components in HOA populations. We used a cross-validation procedure to estimate the best value for the parameter for the number of assigned ancestral populations (K) and found that values from 9 to 14 had the lowest and similar cross-validation errors (Figure S2). (A) The differences in inferred ancestry from K = 9–14 are most pronounced in the HOA for K = 10–12, where two ancestry components that are largely restricted to the HOA appear (the dark purple and dark green components). (B) Surface interpolation of the geographic distribution of eight inferred ancestry components that are relatively unchanging and common to the ADMIXTURE results from K = 10–12. (C) Individual ancestry estimation for HOA populations (with language groups indicated) and surface plots of the changing distributions of the Nilo-Saharan (light blue) and Arabian (brown) ancestry components for K = 10–12. At K = 11, a new HOA-specific ancestry component that we call Ethiopic appears (dark purple) and at K = 12 a second new ancestry component that we call Ethio-Somali (dark green) appears with its highest frequencies in the HOA.

In order to better understand the genetic structure of HOA populations, we analyzed the SNP data using the model-based, maximum likelihood ancestry estimation procedure implemented in the ADMIXTURE software [57] for K values from 2 to 20 (Figure S1). For this analysis, we excluded SNPs in strong linkage disequilibrium, which reduced the main dataset to 16,420 SNPs. We used the cross-validation method encoded in the ADMIXTURE software in an attempt to estimate the optimal number of inferred ancestral components (K) [58]. This cross validation procedure splits the genotype data into partitions and masks (marks as missing) each partition in turn, predicting the genotypes of the masked sites from the remaining unmasked data. For our data, the cross-validation error is minimized at K = 12, but there is little difference in error from K = 9 to K = 14 (Figure S2). For HOA populations, the ADMIXTURE estimates for K = 9–14 fall into three distinct patterns at K = 9–10, K = 11, and K = 12–14 (Figure S1). Here we focus on the ancestral component estimates for K values of 10, 11, and 12 as representative of these three patterns (Figure 2).

There are ten inferred ancestry components (IACs) that are consistent across all three focal K values (Figure 2) and are congruent with published analyses of African and Eurasian population structure [3], [49], [59], [60]. Four IACs are found predominantly in sub-Saharan African populations: (1) one with high frequencies in the Mbuti and Biaka pygmies that is colored pink in the figure; (2) one with high frequencies in Khoesan speaking populations of southern Africa that is colored light purple; (3) one with high frequencies in Niger-Congo speaking populations throughout sub-Saharan Africa that is colored dark blue; and (4) one with high frequencies in Nilo-Saharan speaking populations that is colored light blue. Five IACs are found predominantly in Eurasia: (1) one with high frequencies in Central and South Asian populations that is colored red; (2) one with high frequencies in European populations that is colored light orange; (3) one with its highest frequencies in southern Europe, the Middle East, and Central Asia that is colored dark orange; (4) one with its highest frequencies in Arabian populations that is colored brown; and (5) one with its highest frequencies in Central Asian populations of known East Asian ancestry that is colored grey. The tenth shared IAC is colored light green and predominates in North African populations. This “Maghrebi” IAC has been recovered in previous studies of North African populations and is hypothesized to represent a late Pleistocene migration of non-African ancestors back into Africa [43], [61].

From K = 10 to K = 12, the changes in ADMIXTURE results occur primarily in the HOA, where two new IACs appear at high frequencies (Figure 2). At K = 10 the African ancestry of HOA populations is dominated by the Nilo-Saharan IAC and the non-African ancestry is mostly split between Arabian and Maghrebi IACs. At K = 11 a new African IAC, colored dark purple in the figure, which we refer to as “Ethiopic”, replaces much of the previously Nilo-Saharan attributed ancestry. The Ethiopic IAC reaches its highest frequencies in the Omotic speaking Ari and Wolayta populations, and is present at moderate frequencies in Semitic and Cushitic speaking populations. Pagani et al. [16] previously reported the presence of an equivalent Ethiopia-specific IAC (colored yellow in their Figure 1C). At K = 12 a second new IAC replaces almost all of the Maghrebi and much of the Arabian attributed non-African ancestry. This IAC is colored dark green on the figure and is referenced here as “Ethio-Somali”. This Ethio-Somali IAC is found at its highest frequencies in Cushitic speaking Somali populations and at high frequencies in neighboring Cushitic and Semitic speaking Afar, Amhara, Oromo, and Tygray populations. This IAC was not identified in the source study for the HOA SNP data [16], but Tishkoff and colleagues [59], in an analysis of an independent autosomal microsatellite dataset, did recover an equivalent IAC (calling it “Cushitic”). While this Ethio-Somali IAC is found primarily in Africa, it has clear non-African affinities (Text S1).

Confident determination of the appropriate K value in an ADMIXTURE-like analysis in most human population genomic studies is problematic because the information required to set K *a priori* is unknown. In fact, there is no true K value in most cases because the simultaneous diversification model fit by ADMIXTURE is a poor reflection of human population history. Therefore, rather than take the ADMIXTURE IACs for one of K = 10,11,12 at face value, we used these estimates as hypotheses about the genetic structure of HOA populations and then evaluated these hypotheses in separate analysis.

First, for all focal K values, the ADMIXTURE analysis suggests that many HOA populations have admixture between African and non-African ancestors in their history. To test this, we conducted three formal tests for admixture: the *f_3_*-statistic test, the *D*-statistic test, and a weighted LD test [62], [63]. We found that eight HOA populations (Afar, Amhara, Ari Cultivator, Oromo, Ethiopian Somali, Somali, Tygray, and Wolayta) had statistically significant signals of admixture with non-African populations for all three tests (Tables S2, S3, S4). With this strong support for a history of admixture between African and non-African ancestral populations, the differences among ADMIXTURE IACs across K = 10–12 suggest the following hypotheses for the African ancestry in the HOA:

1. (K = 10) The HOA African ancestry is very similar to that found in neighboring Nilo-Saharan speaking populations.
2. (K = 11,12) There is a distinct, differentiated African ancestry in HOA populations (the Ethiopic IAC).

And the following hypotheses for the non-African ancestry in the HOA:

1. (K = 10,11) HOA populations experienced admixture with one or more non-African populations carrying high levels of the Arabian and Maghrebi IACs along with small amounts of the Eurasian IAC.
2. (K = 12) There is a distinct non-African ancestry in the HOA that constitutes most of the non-African ancestry (the Ethio-Somali IAC).

To evaluate these ADMIXTURE-derived hypotheses, we used the CHROMOPAINTER software [64] to partition the chromosomes of HOA and neighboring populations into segments of African and non-African origin. We then sampled from the painted segments to create composite African and non-African ancestry chromosomes. To ensure that the African and non-African ancestry analyses would be directly comparable, we retained only those SNPs where samples could be generated from both the African and non-African painted segments across all populations, resulting in a dataset that includes 4,340 SNPs (the “4K partitioned” dataset). This dataset includes African and non-African partitioned samples from admixed HOA populations (Afar, Amhara, Ari [Blacksmith and Cultivator combined], Oromo, Somali [Ethiopian Somali and Somali combined], and Tygray) and from admixed Middle Eastern and North African (MENA) samples (Egypt, Mozabite, Palestinian, Yemen) as well as from relatively non-admixed African (Anuak, Gumuz, South Sudanese) and non-African (Bedouin, Druze, Saudi Arabia) populations. Further information on the population selection and ancestry painting methods is detailed in Materials and Methods. We used this 4K partitioned dataset to evaluate hypotheses of gene flow and population structure arising from the ADMIXTURE results.

### African ancestry in the HOA

**Figure 3:**
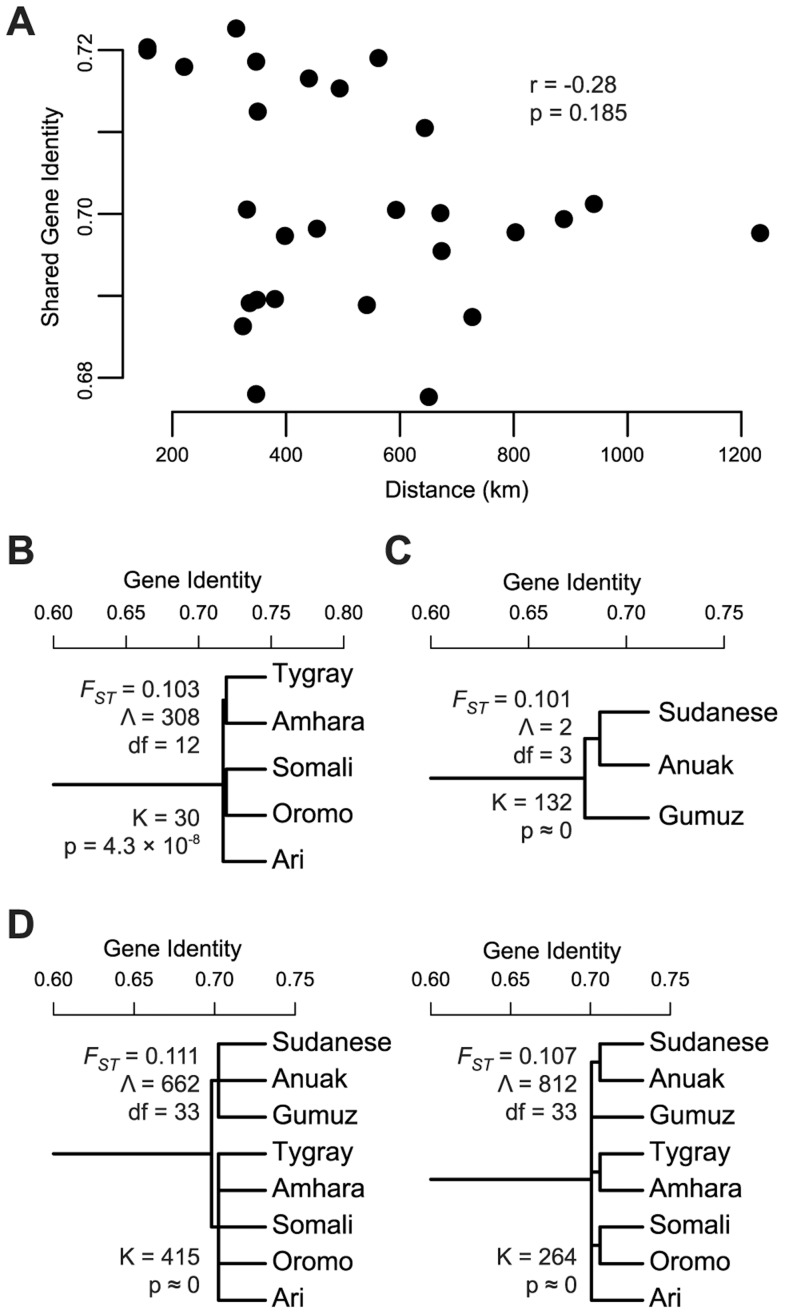
Tests of gene flow and population structure in the African ancestry of HOA populations. Using the African origin partition of the HOA data identified in a chromosome painting analysis, we evaluated the evidence for gene flow with neighboring populations and for population structure within and between the HOA and neighboring populations. (A) Shared gene identity plotted against distance for the HOA populations and the neighboring Anuak, Gumuz, and South Sudanese. (B) Linguistically structured population tree model within the African ancestry partition of HOA populations with the *F_ST_* estimate from this tree model, the goodness-of-fit statistic Λ, and the likelihood ratio test statistic K for the improvement in model fit from the unstructured tree. (C) The linguistically structured population tree model for neighboring Nilo-Saharan language family populations. (D) Structured population tree models for the combined HOA and neighboring populations.

The hypothesis that African ancestry in the HOA is not distinct from that found in neighboring Nilo-Saharan speaking populations (hypothesis 1A above) requires a history of homogenizing inter-population migration or relatively recent common origin. In the case of homogenizing gene flow, a correlation between genetic and geographic distance might be expected, with nearby populations more alike than distant populations. We calculated within and between population gene identity (the probability that two randomly drawn alleles are identical by state) for all the populations included in the 4K partitioned dataset. We then used the between population gene identity estimates among the predominantly Nilo-Saharan ancestry Anuak, Gumuz, South Sudanese, and the African ancestry partition of the Amhara, Ari, Oromo, Somali, and Tygray to test for a relationship between genetic and geographic distance. No significant relationship was recovered (Mantel test, r = −0.28, p = 0.185) (Figure 3A).

Since the pattern of genetic variation in the African ancestry of Sudanese and HOA populations is not a good fit to one model of ongoing gene flow, we tested the hypothesis that there is population substructure within and between HOA and Nilo-Saharan populations using AMOVA [65] and hierarchical population tree models [66], [67]. First, within the HOA we used AMOVA to test for differentiation between linguistic groups – the Omotic speaking Ari, the Semitic speaking Amhara and Tygray, and the Cushitic speaking Oromo and Somali – and found a significant difference (Φ_GT_ = 0.013, p<0.001). We also fit the HOA data to two population tree models, one without substructure and one with linguistically defined subgroups (Figure 3B), and found that the tree with the linguistic groups is a significantly better fit to the data (K = 30, df = 1, p = 4.3×10^−8^). Next, we tested for the presence of linguistically delineated subgroups within the Anuak, Gumuz, and South Sudanese. Most southern Sudanese populations speak languages in the Nilotic branch of the Nilo-Saharan language family and the Anuak language is also a Nilotic language [68]. The Gumuz language is either a highly divergent Nilo-Saharan language or a language isolate [69]. AMOVA reveals a statistically significant difference between these linguistic groups (Φ_GT_ = 0.024, p<0.001) and the population tree with linguistically defined subgroups (Figure 3C) is a significantly better fit to the data than the tree without subgroups (K = 132, df = 1, p≈0). Finally, putting all of the populations together in an AMOVA analysis, we find significant differences between linguistic subgroups at both a macro level (Nilo-Saharan vs Afro-Asiatic) (Φ_GT_ = 0.014, p<0.0001) and a micro level (Nilotic, Gumuz, Omotic, Semitic, Cushitic) (Φ_GT_ = 0.022, p<0.0001). Population tree models with these groupings are a significantly better fit to the data than a tree without subgroups (K = 415 and 264, df = 1, p≈0) (Figure 3D). The tree with the larger subgroups (Nilo-Saharan vs Afro-Asiatic) is a slightly better fit to the data (Λ = 662) than the tree with the smaller subgroups (Λ = 812; smaller Λ values indicate better fit).

These results support the hypothesis from ADMIXTURE K≥11 of a distinct African ancestry with a long history in differentiated HOA populations (hypothesis 1B above) over the hypothesis from ADMIXTURE K≤10 that African ancestry in the HOA is not substantially differentiated from that found in neighboring populations (hypothesis 1A). In fact, our results suggest a rather more complicated history for these regional populations. Studies of further population samples from ethnic groups in and near the western and southern edges of the Ethiopian escarpment are sure to be interesting.

### Non-African ancestry in the HOA

**Figure 4:**
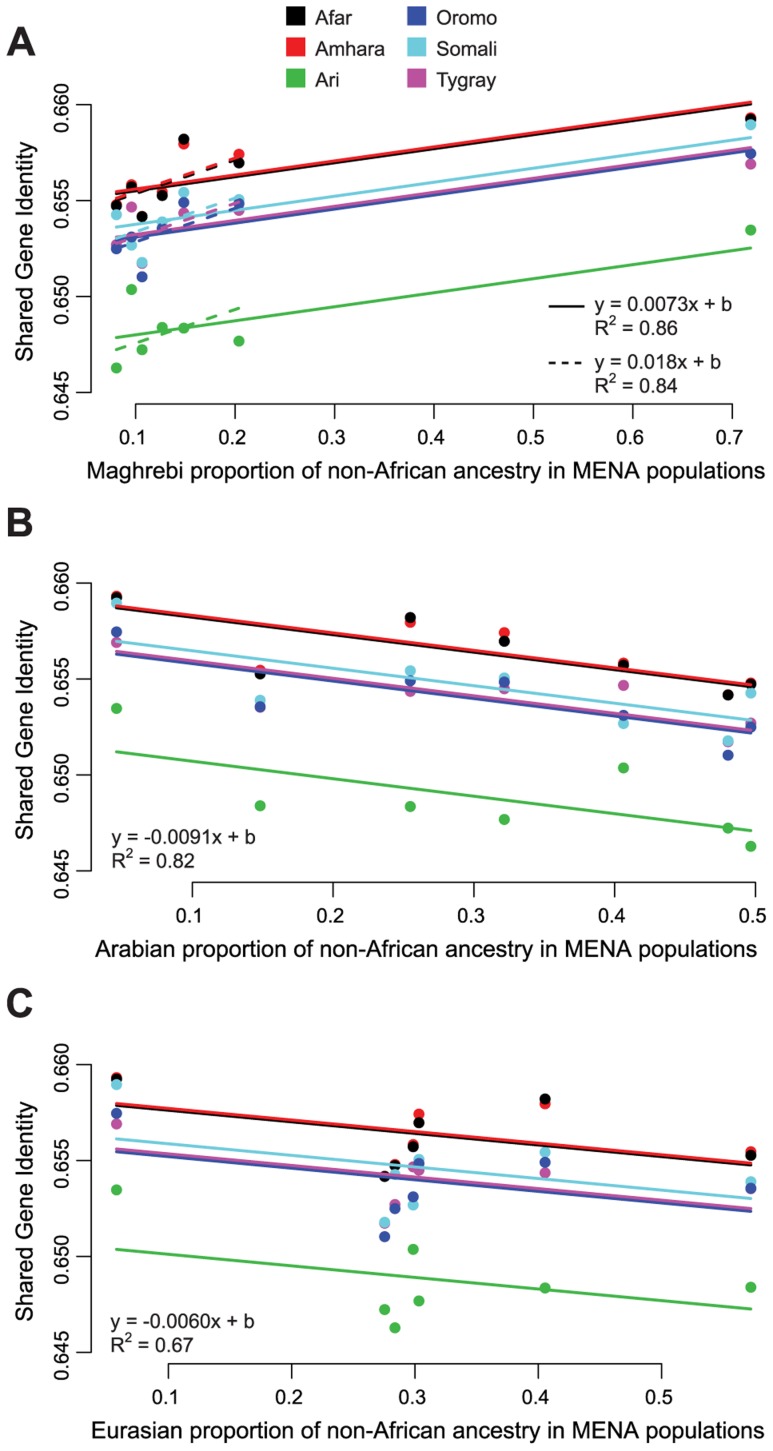
Relationship between non-African ADMIXTURE ancestry components and shared gene identity between HOA and MENA populations. ADMIXTURE results for K = 10,11 suggest that the non-African ancestry in HOA populations is indistinguishable from the “Maghrebi,” “Arabian,” and “Eurasian” ancestry components found in MENA populations. If this is a correct inference, then the shared gene identity of HOA populations should be higher with MENA populations with higher proportions of these ancestries. (A) There is significant positive relationship between shared gene identity and the proportion of Maghrebi ancestry in MENA populations. While there is variation across HOA populations in the overall shared gene identity (different intercepts for each population), the magnitude of the relationship is consistent (adding varying slopes did not significantly improve the model fit). This relationship holds whether or not the Mozabite (a high Maghrebi ancestry outlier) are included in the model. However, contrary to expectations, both the Arabian (B) and Eurasian (C) ancestry components showed a reduction in shared gene identity as the representation of these ancestry components in MENA populations increased.

The ADMIXTURE-derived hypothesis that non-African ancestry in the HOA derives from admixture with a population or populations with high levels of the Arabian and Maghrebi IACs and some of the Eurasian IAC (hypothesis 2A above) suggests that HOA populations should have higher levels of shared gene identity with populations with higher proportions of those ancestries. To evaluate this prediction, we examined the relationship between shared gene identity and the ADMIXTURE-estimated proportion of the Arabian, Eurasian, and Maghrebi IACs in MENA population samples for each of the non-African ancestry partitions of the admixed HOA populations using varying intercepts linear models. Only the Maghrebi IAC analysis shows the expected relationship: shared gene identity between HOA and MENA populations increases as the proportion of Maghrebi ancestry increases (Figure 4A). Contrary to expectations, shared gene identity decreases between HOA populations and MENA populations as the proportion of the Arabian IAC (Figure 4B) and the Eurasian IAC (Figure 4C) increases.

**Figure 5:**
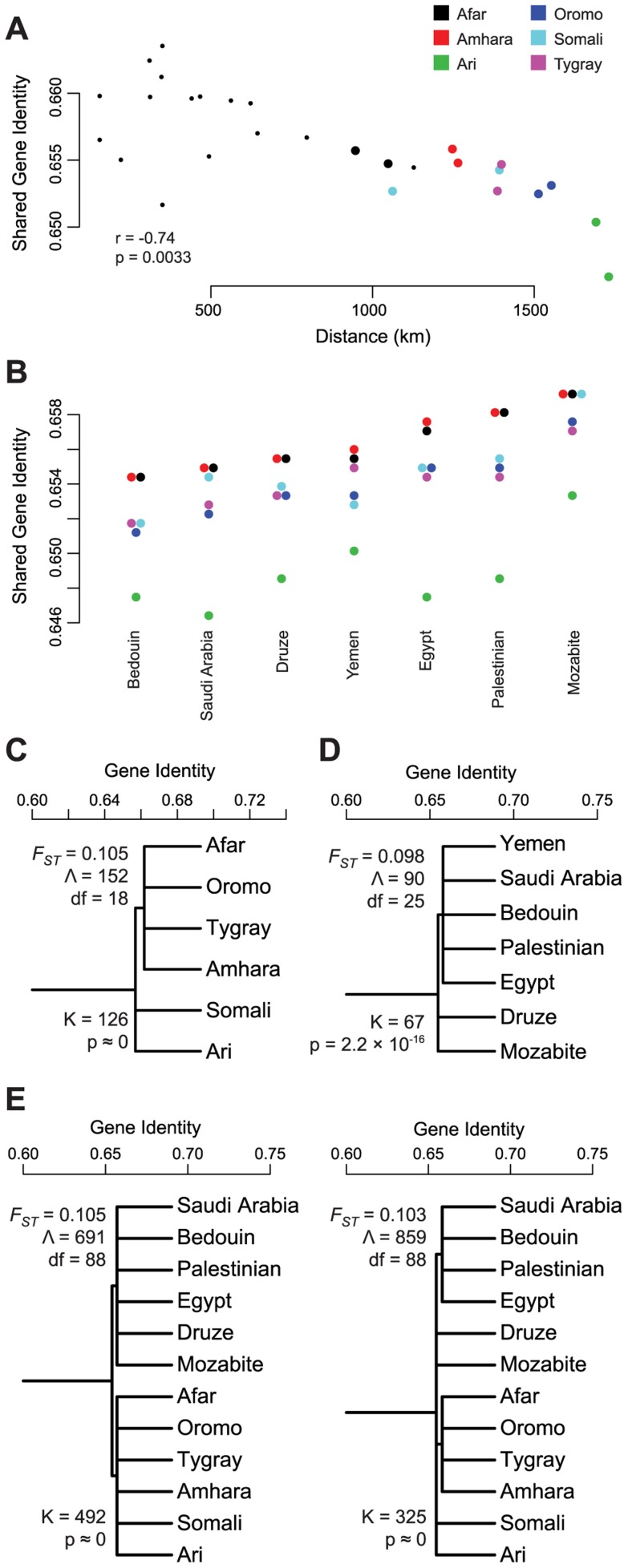
Tests of gene flow and population structure in the non-African ancestry of HOA populations. Using the non-African origin partition of the HOA data identified in a chromosome painting analysis, we evaluated the evidence for gene flow with MENA populations and for population structure within and between the HOA and MENA populations. (A) The only clear and statistically significant pattern of decreasing gene identity with geographic distance was between HOA populations and the Yemen and Saudi Arabia populations on the Arabian peninsula as evaluated by a Mantel test. This relationship only held for “as the crow flies” geographic distances; the relationship disappears using a waypoint through Egypt (Figure S3). (B) Shared gene identity between the non-African ancestry partition of HOA populations and MENA populations presented in increasing order. (C) Structured population tree model within the non-African ancestry partition of HOA populations with the *F_ST_* estimate from this tree model, the goodness-of-fit statistic Λ, and the likelihood ratio test statistic K for the improvement in model fit from the unstructured tree. (D) Structured population tree model within the non-African ancestry partition of MENA populations. (E) Structured population tree models for the non-African ancestry partitions of both HOA and MENA populations. Both are significantly better fits to the data than the unstructured tree and the regional structure (HOA vs MENA) is a slightly better fit to the data as measured by the goodness-of-fit Λ statistic.

Next, we looked for evidence for extended inter-population gene flow in the correlation of geographic distance and shared gene identity. We found no relationship between geographic and genetic distance within either HOA or MENA populations. We then examined this relationship for HOA populations to North African (Egypt, Mozabite), Levantine (Bedouin, Druze, Palestinian), and Arabian (Saudi Arabia, Yemen) populations (Figure S3). For North Africa and Arabia, we calculated both straight-line distances and distances involving a waypoint through Egypt. The only group for which there is a clear gradient of genetic similarity decreasing with geographic distance is for the straight-line distances with Arabian populations (Mantel test, r = −0.74, p = 0.0033) (Figure 5A). This relationship between genetic and geographic distance between HOA and Arabian populations might support a hypothesis of long-term equilibrium gene flow among these populations in an isolation-by-distance model. However, if this hypothesis were true, we would expect the highest levels of pairwise gene identity to be between HOA and Arabian populations, but this is not the case. The highest levels of shared gene identity are between HOA populations and the Levantine Palestinian and the North African Mozabite population samples (Figure 5B). Thus, it is more likely that the genetic-geographic HOA-Arabia distance gradient reflects secondary admixture of Arabian migrants into HOA populations already carrying substantial non-African ancestry or already admixed HOA populations sending migrants into Arabian populations.

While these results suggest some history of gene flow between HOA populations and MENA populations, there is no simple pattern that emerges. In order to better understand the partitioning of genetic variation among these populations, we tested for population substructure within and between HOA and MENA populations using AMOVA and hierarchical population tree models. First, within the HOA, we tested for linguistically defined substructure between Cushitic, Semitic, and Omotic speaking populations, but found no significant differentiation (Φ_GT_ = 0.010, p = 0.066). We then used the ADMIXTURE results to inform subgroup formation. At K = 12, the Amhara, Tygray, Oromo, and Afar all have similar proportions of non-African ancestries that differ from that seen in the Ari and Somali (Figure 2). This observation suggests a geographical structuring between the Amhara, Tygray, Oromo, and Afar in the Ethiopian highlands, the Somali in eastern Ethiopia and the Somalia lowlands, and the Ari in the southwestern Ethiopian Rift. AMOVA of these three population groups reveals significant between group differentiation (Φ_GT_ = 0.017, p<0.0001). In addition, the population tree with these geographic subgroups (Figure 5C) is a significantly better fit to the data than the tree without subgroups (K = 126, df = 1, p≈0). Within MENA populations, linguistic subgroups cannot be defined, so we tested several historic/geographic groupings. Between population differentiation was maximized in the AMOVA analysis with three subgroups: the northwest African Mozabite; the ethnic and religious isolate Druze; and the populations with histories entwined with the development and expansion of Islam - the Egyptians, Palestinians, Bedouin, Saudi Arabians, and Yemeni. For this set of subgroups, between population differentiation was statistically significant (Φ_GT_ = 0.011, p<0.0001) and the population tree with these subgroups (Figure 5D) is a significantly better fit to the data than the tree without subgroups (K = 67, df = 1, p = 3.3×10^−16^). Finally, putting all of the populations together in an AMOVA analysis, we find significant differences between HOA and MENA subgroups at both a macro level (HOA vs MENA) (Φ_GT_ = 0.014, p<0.0001) and a micro level (all of the individual subgroups identified above) (Φ_GT_ = 0.016, p<0.0001). Population tree models for both the simple (HOA vs MENA) and more complex (all individual regional subpopulations) groups are a significantly better fit to the data than a tree without subgroups (Figure 5E). As measured by the goodness-of-fit statistic Λ of Long and Kittles [67], the simple HOA vs MENA structure is the better first level structure fit to the data than the more complex structure with six different subgroups.

Even though there is strong evidence for admixture between HOA and MENA populations, there is also clearly detectable substructure both within and between the HOA and the Middle East and North Africa. If the majority of the non-African ancestry in the HOA had entered during the last few thousand years (hypothesis 2A above), then population groups should be less differentiated within the HOA then within MENA samples. This expectation is reinforced by the smaller geographic area of sampling within the HOA when compared to the geographic spread of the MENA samples. However, what we observe is that population groups within the HOA are more differentiated (Φ_GT_ = 0.017; *F*_ST_ = 0.105) than population groups across the MENA region (Φ_GT_ = 0.011; *F*_ST_ = 0.098). All together, these results offer greater support to the hypothesis from ADMIXTURE K≥12 that there is a distinct non-African ancestry in the HOA that is well-differentiated from the non-African ancestry in neighboring Middle Eastern and North African populations (hypothesis 2B).

### Timing of non-African admixture in the HOA

We used two methods that model the pattern of linkage disequilibrium (LD) expected to result from admixture to estimate the date of admixture for all study populations in which we found statistically significant signals of admixture: ROLLOFF [62], [70] and ALDER [63]. Our estimates are broadly compatible with the dates previously calculated for these same population samples [16], [17]. Using the HapMap YRI (Yoruba) and CEU (Utah residents with Northern and Western European ancestry) as reference populations, Pagani et al. [16] calculated ROLLOFF admixture dates ranging between 2,000 and 3,000 years ago. Pickrell et al. [17] calculated ALDER admixture date estimates for these populations between about 2,500 and 3,500 years ago, with some experiencing secondary admixture between 100 and 300 years ago. Across the entire set of reference populations that we used, our ROLLOFF estimates range from 2,200 to 4,700 years ago, and our single-admixture ALDER estimates are somewhat younger, ranging from 1,000 to 4,300 years ago (Table 1). Following Pickrell et al. [17], we compared the fit of single and dual admixture histories from ALDER in HOA populations and found, in agreement with their results, strong evidence for two admixture events in the Amhara and Oromo (Table 1).

**Table 1:** Estimates of admixture dates in Horn of Africa populations (ka).^1^

Population	African	non-African	ROLLOFF	ALDER^2^	ALDER^3^
Afar	Yoruba (YRI)	CEU	3.6±0.5	3.2±1.0†	4.4/0.1
	Sudanese	Sardinian	4.7±0.6	4.3±1.0†	
	Sudanese	Turkey	4.7±0.6	3.6±0.8*	
Amhara	Yoruba (YRI)	CEU	2.8±0.2	2.6±0.4*	3.0/0.3
	Sudanese	Sardinian	3.5±0.2	2.0±0.4*	3.4/0.4
	Juhoansi	Sardinian	2.8±0.3	2.0±0.5	3.3/0.5
Ari Cultivator	Yoruba (YRI)	CEU	3.2±0.4	4.1±0.5†	
	Juhoansi	Sardinian	3.4±0.5	3.2±0.6	
Oromo	Yoruba (YRI)	CEU	2.7±0.3	1.0±0.8†	2.6/0.1
	Juhoansi	Sardinian	3.1±0.4	1.7±0.5†	3.3/0.1
	Sudanese	Basque	2.9±0.3	2.0±0.4*	3.1/0.3
Ethiopian Somali	Yoruba (YRI)	CEU	3.7±0.5	3.8±1.4†	
	Sudanese	Sardinian	4.2±0.5	3.1±1.1†	
	Juhoansi	Tuscan (TSI)	3.7±0.4	4.0±0.6	
Somali	Yoruba (YRI)	CEU	2.9±0.4	2.6±0.4†	
	Sudanese	Sardinian	3.9±0.4	2.6±0.6†	
	Juhoansi	Basque	3.7±0.4	3.1±0.4*	
Tygray	Yoruba (YRI)	CEU	3.6±0.3	1.7±0.4†	
	Sudanese	Sardinian	3.6±0.4	2.5±0.6	
Wolayta	Yoruba (YRI)	CEU	3.1±0.8	1.3±0.4	
	Juhoansi	Sardinian	2.2±0.6	1.1±0.3†	

1 Using 30 years per generation.

2 Single admixture fit.

3 Two admixtures fits that are significantly better than the single admixture fit.

† ALDER test for admixture not significant.

* ALDER reports inconsistent decay rates.

These relatively recent dates are not consistent with our results showing a distinct, differentiated non-African ancestry in the HOA. To be sure, the greater shared gene identity between HOA populations and MENA populations with higher proportions of the Maghrebi IAC (Figure 4A) and the observed genetic-geographic correlation with Arabian populations (Figure 4B) are supportive of some relatively recent admixture. However, the high population differentiation found in the AMOVA and population tree analyses (Figure 5) suggests that admixture within the last few thousand years is a poor explanation for the majority of non-African ancestry in the HOA.

In order to seriously entertain the hypothesis that most of the non-African ancestry in the HOA predates the last few thousand years, we must understand why the ROLLOFF and ALDER admixture date estimates are all relatively recent. To do so, we performed simulation tests of ROLLOFF and ALDER in episodic admixture scenarios. To start, we simulated two episodes of admixture, with the first (earliest) episode between 50 and 200 generations ago (1,500–6,000 years using a 30 year generation time) and the second (more recent) episode at either 10 or 30 generations ago (300 or 900 years). We found a strong bias towards the most recent admixture date for both ROLLOFF and single-episode ALDER (Figure S4). To investigate the importance of the relative contribution of the first and second admixtures, we simulated both equal (10% first, 10% second) and unequal (25% first, 10% second) admixture proportions. Variation in the relative contribution of the first and second admixture episodes had a much greater effect on the ROLLOFF estimates than on the ALDER estimates (Figure S4). The stronger bias towards the date of the most recent admixture in the ALDER results is actually desirable, as this tendency makes the ALDER results much more interpretable.

To get an intuition for how ROLLOFF and ALDER perform for truly ancient admixture followed by more recent admixture, we simulated 50% admixture between 1,500 and 35,000 years ago, followed by 10% admixture at 900 years ago (Figure S5). As in the first simulation results, the ALDER estimate is almost always a reasonable estimate of the most recent admixture. If these results hold more generally, then a ROLLOFF estimate many times greater than the ALDER estimate might indicate ancient admixture; however, we are wary of over-generalizing from these data because the relationship between ROLLOFF and ALDER estimates appears to be similar for a broad range of admixture scenarios. Unfortunately, from the ROLLOFF and ALDER estimates alone, it is not possible to say when admixture started, whether it was continuous or successive, if there were multiple sources, how long it lasted, or if there was variation in admixture proportions over time [62]. What is clear is that admixture date estimates from either ROLLOFF or ALDER within the last few thousand years do not preclude the possibility of earlier episodes of admixture.

In order to evaluate the hypothesis that there were two or more distinct episodes of non-African admixture in the HOA, with the Ethio-Somali admixture occurring during an earlier episode, we conducted four analyses. First, we looked at the distribution of IACs among HOA populations from the K = 12 ADMIXTURE results. If there have been successive episodes of admixture into a culturally diverse region, we expect that different populations will have different histories of admixture [60], [71]. Over time, admixed ancestry will be transmitted throughout the region via intra-regional gene flow, including into populations that have no history of direct admixture. If admixture predates modern population divisions, contemporary populations may carry admixed ancestry from a common admixed ancestor. In the HOA, this suggests that the Ethiopic IAC has the deepest roots in the region, as it is present at appreciable frequencies in all populations (Figure 2, Table 2). Next, the Nilo-Saharan IAC is found in all but the Ari Blacksmiths. The Ethio-Somali IAC has the third broadest distribution, and is found in all Cushitic and Semitic speaking populations as well as the Omotic speaking Wolayta and Ari Cultivators, but not the Ari Blacksmiths. Arabian, Eurasian, and Niger-Congo IACs have successively narrower distributions in the HOA. Based on this distribution of IACs across HOA populations, the most parsimonious order of origin in or migration into the region is Ethiopic – Nilo-Saharan – Ethio-Somali – Arabian – Eurasian – Niger-Congo, with the Nilo-Saharan and Niger-Congo gene flow probably coming from the west/southwest and the Ethio-Somali, Arabian, and Eurasian IACs likely arriving from the east/north.

**Table 2:** Estimated mean proportion of ancestry (greater than 5%) in Horn of Africa populations.^1^

	Nilo-Saharan speaking		Cushitic speaking				Semitic speaking		Omotic speaking		
	Anuak	Gumuz	Afar	Oromo	Ethiopian Somali	Somali	Amhara	Tygray	Wolayta	Ari Cultivator	Ari Blacksmith
Niger-Congo	0.11										
Nilo-Saharan	0.75	0.59	0.20	0.19	0.22	0.23	0.16	0.17	0.15	0.10	
Ethiopic	0.07	0.35	0.08	0.21	0.08	0.06	0.16	0.12	0.35	0.63	0.94
Ethio-Somali			0.43	0.32	0.53	0.57	0.35	0.35	0.27	0.17	
Arabian			0.12	0.12			0.16	0.16	0.08		
Eurasian							0.07	0.08			

1 None of the Horn of Africa populations have 5% or greater ancestry from Khoesan, Pygmy, Maghrebi, European, South Asian, or East Asian ancestral populations, so these ancestries are not shown in the table.

Second, at the time of admixture, there would be a great deal of variation among individuals in the amount of ancestry from introgressing populations. After admixture ends, there will be a decrease in variation among individuals in the amount of admixed ancestry over time [72]. We calculated the coefficient of variation for all non-African IACs present above 5% in admixed HOA populations (Table 3). While all significantly admixed HOA populations have at least 5% of the Ethio-Somali IAC, only in a few populations are the Eurasian and Arabian IACs present above 5% (Table 2). In all cases, the coefficient of variation for the Eurasian and Arabian IACs is 2–5 times greater than that for the Ethio-Somali IAC, suggesting that the Ethio-Somali admixture predates the Eurasian and Arabian admixture. The largest coefficient of variation found for the Ethio-Somali IAC is in the Ari Cultivators. As the Ari Blacksmiths have negligible Ethio-Somali ancestry, it seems most likely that the Ari Cultivators are the descendents of a more recent admixture between a population like the Ari Blacksmiths and some other HOA population (i.e. the Ethio-Somali ancestry in the Ari Cultivators is likely to substantially postdate the initial entry of this ancestry into the region).

**Table 3:** Coefficients of variation for Ethio-Somali and non-African ancestry components present above 5%.

Population	Ethio-Somali	Arabian	Eurasian
Afar	0.46	1.16	-
Amhara	0.70	1.46	3.06
Ari Cultivator	1.04	-	-
Oromo	0.76	2.19	-
Ethiopian Somali	0.42	-	-
Somali	0.39	-	-
Tygray	0.62	1.02	2.55
Wolayta	0.34	1.66	-

Third, we estimated divergence times among the IACs (Table 4) using a simple model of the expected relationship among *F*_ST_, effective population size, and divergence time [73], similar to analyses conducted in prior studies of North African and Levantine samples [43], [60]. The most recent divergence date estimates for the Ethio-Somali ancestral population are with the Maghrebi and Arabian ancestral populations at 23 and 25 ka. Among the many assumptions made for this calculation is that the pairwise *F*_ST_ values for the ADMIXTURE IACs reflect post-divergence population isolation and could be used to construct a bifurcating population tree that is a good fit for the data. If the population tree model is generally valid, but there has been some post-divergence migration, then the fit of the data to the tree model will not be good and the true divergence date would have been earlier than what we estimate here. When we evaluate the fit of the non-African IACs to a population tree model (Figure S6) using the goodness-of-fit statistic Λ of Long and Kittles [67], we find that the data deviate significantly from a good fit (Λ = 1064, df = 15, p≈0) and therefore the dates calculated here assuming a population divergence model are likely to be underestimates.

**Table 4:** Minimum time of divergence of ADMIXTURE inferred ancestry components (ka).

	Niger-Congo	Nilo-Saharan	Ethiopic	Ethio-Somali	Maghrebi	Arabian	Eurasian
**Khoesan**	34	39	40	**66**	66	73	75
**Pygmy**	26	31	34	**49**	59	67	68
**Niger-Congo**	-	15	24	**34**	43	52	54
**Nilo-Saharan**	15	-	22	**33**	43	51	53
**Ethiopic**	24	22	-	**31**	38	44	46
**Ethio-Somali**	34	33	31	**-**	23	26	31
**Maghrebi**	43	43	38	**23**	-	19	21
**Arabian**	52	51	44	**26**	19	-	18
**Eurasian**	54	53	46	**31**	21	18	-
**European**	58	57	50	**33**	19	19	14
**South Asian**	50	49	44	**35**	29	27	24
**East Asian**	52	51	46	**37**	31	30	26

An alternative interpretation of the *F*_ST_ estimates is also possible. Wright originally formulated *F*_ST_ as a measure of differentiation between populations that is the result of an equilibrium between the opposing forces of gene flow and genetic drift [73]. In this formulation, the measured populations have never been completely isolated from each other and it would be inappropriate to attempt to calculate a divergence date from the *F*_ST_ value. The extremely poor fit of the IAC data to a bifurcating population tree model suggests that a population history without population isolation is also a possibility. Overall, the pairwise *F*_ST_ estimates for the IACs suggest that either the ancestral Ethio-Somali population had begun to differentiate from other non-African populations by at least 23 ka or that the ancestral Ethio-Somali population has never been completely isolated from other non-African populations. In either case, these data do not indicate when this population arrived in the HOA. When complete genome sequences become available for HOA, North African, and Middle Eastern populations, it should be possible to obtain better estimates using new methods being developed for unbiased sequence data, such as those based on the site frequency spectrum [74].

Fourth, a unique East African lactase persistence allele is found at its highest frequency in the Maasai [75] who have about 21% Ethio-Somali ancestry (Table S5). This lactase persistence allele is different from the alleles associated with lactase persistence in Europe [76], [77] or Arabia [78], [79], and likely arose during the last 7,000 years [75]. The Maasai do not have the Arabian lactase persistence allele, which is estimated to have originated about 4,000 years ago (95% CI: 250–27,575) and is present at high frequencies in Arabian populations (>50%) [78], [79]. This Arabian allele is also almost absent in the Somali (1.6%) [79], which further supports our hypothesis that gene flow from Arabia within the last few thousand years cannot explain the non-African ancestry in HOA populations.

In summary, while LD-based methods estimate the time of non-African admixture in HOA populations to be within the last few thousand years, all sampled neighboring populations in North Africa or the Middle East are substantially differentiated from the non-African ancestry in the HOA. Based on this discrepancy, we undertook a closer examination of the properties of the LD-based ROLLOFF and ALDER admixture time estimation methods and found that earlier episodes of admixture are largely masked by more recent admixture events. Therefore, the admixture dates that are found within the last few thousand years do not falsify the hypothesis that the Ethio-Somali IAC arrived in the HOA during an earlier admixture event. (The ALDER/ROLLOFF simulation results do not directly support the hypothesis of earlier admixture, but they do show that earlier admixture cannot be excluded). The key lines of evidence that support a hypothesis of earlier admixture are that the Ethio-Somali IAC is broadly distributed across almost all ethnic groups in the HOA, consistent with an early entry into the region; that there is less inter-individual variance in Ethio-Somali IAC among individuals within ethnic groups than in Arabian or Eurasian IACs, suggesting that Ethio-Somali admixture predates the Arabian and Eurasian admixture; that the Ethio-Somali IAC is estimated to have diverged from all other non-African IACs by at least 23 ka; and that the Ethio-Somali IAC does not contain the unique Arabian lactase persistence allele that arose about 4 ka. In combination, these data suggest that the Ethio-Somali ancestors admixed with African-origin HOA ancestors sometime after 23 ka, but before the Middle Eastern admixture during the last few thousand years.

### Non-genetic evidence for the timing of the Ethio-Somali back-to-Africa migration

Agriculture was established in the HOA by at least 7 ka [14], [80], which suggests that local population densities were likely to have been relatively high from that time forwards. An external migration that occurred recently leading to 30–60% total genome-wide representation into pre-existing agricultural populations (Table S5) would require large or sustained population movements, which is not supported by either the historical or archaeological record [4], [14]. The Ethio-Somali ancestry is more likely to have arrived during an earlier hunter-gatherer phase, when a smaller migration could make a significant contribution. As a point of reference, the slave trade into North Africa and the Middle East of over 11 million sub-Saharan Africans over the last 1,400 years [81] has led to a maximum of 30% total African ancestry in these populations.

Paleoclimate data offer some information on time ranges when human migration back-to-Africa would be most likely to succeed. During arid periods in North Africa and the Middle East, most plausible routes into Africa experienced desertification, reducing the likelihood of successful migration. In our time frame of interest, there have been two major peaks of aridity in the region, the Last Glacial Maximum (LGM: ∼21.5 ka) and the Younger Dryas (YD: ∼12.5 ka), during which successful human migrations would not have been likely [82]–[86]. Since the end of the YD there have been fluctuations of arid and wet phases, but no arid periods as extreme or long lasting as these earlier two intervals [82]. Thus, if the Ethio-Somali ancestors diverged from all other non-African populations by 23 ka and were present in the HOA before the advent of HOA agriculture at around 7 ka [80], then there are three possible window of migration: post-YD, between the YD and the LGM, and pre-LGM. Because agriculturalist populations were expanding rapidly in the Middle East beginning about 12 ka and early agriculture in the HOA has an independent origin [80], the earlier YD-LGM and pre-LGM windows are favored.

There is abundant archaeological material in the HOA dating to between 5 and 30 ka, but most of the published literature is descriptions of surface surveys or test excavations [87], [88]. More extensive investigations have focused on patterns of resource utilization [89]–[92], a key archaeological research goal, but less helpful for identifying cultural or biological affinities of early HOA populations. Contemporary HOA populations have occasionally been included in craniometric or dental studies of the biological affinities of ancient North Africans and Egyptians [93]–[95], but very little comparative analysis is available for the few prehistoric HOA skeletal collections [89], [96]. One possible indication of ancient Ethio-Somali admixture might be found in studies of Late Pleistocene Nubians (∼12 ka) from the Nile River Valley, who have been variously interpreted as sharing affinities with contemporaneous North African Iberomaurusians [97] and with sub-Saharan Africans [98]. Admixture of Ethio-Somali ancestors with African-origin populations in this region might explain these divergent interpretations of this Late Pleistocene Nubian population.

### Relationship to the North African back-to-Africa migration

Like the Ethio-Somali, the Maghrebi IAC in North African populations derives from a early back-to-Africa migration [34], [43], [61], [99]–[102]. Studies of North African populations reveal a complex layered history of admixture in North Africa, with an inferred pre-Last Glacial Maximum settlement of North Africa by a non-African population followed by gene flow from European, Middle Eastern, and sub-Saharan African populations dating from the end of the LGM to the recent past [43], [103]–[105].

A single prehistoric migration of both the Maghrebi and the Ethio-Somali back into Africa is the most parsimonious hypothesis. That is, a common ancestral population migrated into northeast Africa through the Sinai and then split into two, with one branch continuing west across North Africa and the other heading south into the HOA. For the Ethio-Somali, the lowest *F*_ST_ value from the ADMIXTURE estimated ancestral allele frequencies is with the Maghrebi (Text S1), which is consistent with a common origin hypothesis. In contrast, the Maghrebi component has lower *F*_ST_ values with Arabian, European, and Eurasian ancestral populations than with the Ethio-Somali, which suggests that the Maghrebi diverged most recently from those populations, and might indicate separate back-to-Africa migrations for the Ethio-Somali and the Maghrebi. Unfortunately, the *F*_ST_ estimates alone are not robust enough to distinguish between single or separate back-to-Africa migrations. While the *F*_ST_ estimates for the ancestral populations are, in theory, free of confounding admixture, they derive from a simplified model of population history that is known to be inaccurate (simultaneous divergence) and are all assumed to be in Hardy-Weinberg equilibrium [57], [106]. As a result, fine-scale differences in pairwise *F*_ST_ among ancestral populations should be interpreted with care.

Mitochondrial M1 and U6 lineages – sub-clades of mitochondrial haplogroups that are otherwise found only in Eurasian populations – are found both in North Africa and the HOA [34]. U6 has its highest frequencies and diversity in Northwest Africa and M1 has its highest frequencies and diversity in the HOA. The differing representation of deeply diverging M1 and U6 mitochondrial lineages in North Africa and the HOA shows that these regions have exchanged few female migrants since approximately 20 ka [36]. While these mitochondrial data further support our hypothesis that most of the non-African ancestry in the HOA has an ancient origin, we still cannot distinguish between single or separate migrations of the Maghrebi and Ethio-Somali back-to-Africa. If we could identify the geographical origins of both M1 and U6 and if these lineages originated in the same area, then a common migration hypothesis would seem more likely. The geographical origin of a mitochondrial clade is usually inferred from the presence of diverse early branching lineages within a region. To date, no region has been identified with a diversity of early branching lineages of either M1 or U6. Given the exclusively Eurasian distribution of the larger M and U haplogroups, it is generally inferred that M1 and U6 originated outside of Africa [34], [35], [100] but since all other early branches of M1 and U6 appear to have gone extinct, it is not possible to specify their location of origin. Most recently, Pennarun and colleagues [36] found that sub-lineages within U6 began diversifying in North Africa about 10,000 years before M1 sub-lineages began diversifying in the HOA (∼30 ka vs. ∼20 ka). This difference in coalescence times might be taken as evidence for separate migrations, but could also be explained by smaller population sizes in the HOA ancestors between 30 and 20 ka following a common migration.

### Summary and implications

We find that most of the non-African ancestry in the HOA can be assigned to a distinct non-African origin Ethio-Somali ancestry component, which is found at its highest frequencies in Cushitic and Semitic speaking HOA populations (Table 2, Figure 2). In addition to verifying that most HOA populations have substantial non-African ancestry, which is not controversial [11]–[14], [16], we argue that the non-African origin Ethio-Somali ancestry in the HOA is most likely pre-agricultural. In combination with the genomic evidence for a pre-agricultural back-to-Africa migration into North Africa [43], [61] and inference of pre-agricultural migrations in and out-of-Africa from mitochondrial and Y chromosome data [13], [32]–[37], [47], [99]–[102], these results contribute to a growing body of evidence for migrations of human populations in and out of Africa throughout prehistory [5]–[7] and suggests that human hunter-gatherer populations were much more dynamic than commonly assumed.

We close with a provisional linguistic hypothesis. The proto-Afro-Asiatic speakers are thought to have lived either in the area of the Levant or in east/northeast Africa [8], [107], [108]. Proponents of the Levantine origin of Afro-Asiatic tie the dispersal and differentiation of this language group to the development of agriculture in the Levant beginning around 12 ka [8], [109], [110]. In the African-origins model, the original diversification of the Afro-Asiatic languages is pre-agricultural, with the source population living in the central Nile valley, the African Red Sea hills, or the HOA [108], [111]. In this model, later diversification and expansion within particular Afro-Asiatic language groups may be associated with agricultural expansions and transmissions, but the deep diversification of the group is pre-agricultural. We hypothesize that a population with substantial Ethio-Somali ancestry could be the proto-Afro-Asiatic speakers. A later migration of a subset of this population back to the Levant before 6 ka would account for a Levantine origin of the Semitic languages [18] and the relatively even distribution of around 7% Ethio-Somali ancestry in all sampled Levantine populations (Table S6). Later migration from Arabia into the HOA beginning around 3 ka would explain the origin of the Ethiosemitic languages at this time [18], the presence of greater Arabian and Eurasian ancestry in the Semitic speaking populations of the HOA (Table 2, S6), and ROLLOFF/ALDER estimates of admixture in HOA populations between 1–5 ka (Table 1).

## Materials and Methods

### Ethics statement

Saliva samples were collected in Yemen in 2007 with informed consent under Western IRB approval, Olympia, WA. Subsequent analysis of anonymized SNP data was approved by the Lehman College IRB.

### Genotyping of new Yemeni samples

Sixty-four Yemeni, chosen to represent all geographic regions of the country, were selected for SNP genotyping. Genomic DNA was extracted from saliva samples (DNA Genotek Oragene collectors) using the manufacturer's protocol. This DNA was genotyped using the Illumina 370k SNP chip by the University of Florida Interdisciplinary Center for Biotechnology Research Core Facility following the manufacturer's protocols. These new data are available from the Dryad Digital Repository (http://dx.doi.org/10.5061/dryad.d9s74) [112].

### Data sets

We merged genome-wide SNP data from the HOA [16] with the new Yemeni data and other published data from the Middle East [48], North Africa [43], Qatar [50], southern Africa [51], west Africa [49], the HapMap3 project [52], and the Human Genome Diversity Project [53] using PLINK version 1.07 [113]. We excluded symmetric SNPs and SNPs and individuals with greater than 10% missing data. All known and inferred relatives were removed from the HapMap3 and HGDP data [114], [115]. We then estimated kinship coefficients across all remaining individuals in all included populations using the “robust” algorithm, which is tolerant of population structure, in the KING software [116]. For all sets estimated to be second degree or closer relatives, we removed the individual(s) that would maximize the number of included individuals.

After pre-processing, the main dataset included 2,194 individuals from 81 populations for 16,766 SNPs (Table S1). We generated the linkage map for this dataset using the online map interpolator from the Rutgers second-generation combined linkage-physical map [117]. This dataset include some markers in strong linkage disequilibrium (LD), which is required for some of the analyses we conducted, but can bias other methods. For the methods that can be confounded by high levels of LD, we randomly excluded one of every pair of SNPs having pairwise genotypic correlation greater than 0.5 within a sliding 50 SNP window. After this exclusion, the “reduced-LD” dataset had 16,420 SNPs.

Many methods are known to perform better with more SNPs, especially those based on patterns of LD. To ensure that the estimates using these methods from our main dataset are reliable, we created two additional verification datasets with reduced population representation, which allows for greater overlap of mutually typed SNPs across studies. The “90K” dataset includes data for 91,101 SNPs from HOA, HapMap3, HGDP, and North Africa populations. The “260K” dataset includes data for 259,257 SNPs from the HOA, HapMap3, HGDP, southern Africa, and selected West Asian populations (see Table S1 for populations in the 90K and 260K datasets). All of the procedures described above for the main datasets were followed.

### Population structure

Multidimensional scaling (MDS) was performed upon a genome wide matrix of identity by state (IBS) for all individual pairs in the reduced-LD dataset using PLINK [113]. For each increase in K from 2 to 5, there were substantial changes in reduced stress, but not for K greater than 5, so the IBS matrices were projected to 5-dimensional space. We inferred genetic structure and estimated admixture proportions in the reduced-LD dataset using ADMIXTURE [57]. Ancestry proportions were estimated for K values ranging from 2 to 20, and cross-validation error was calculated for each value of K. The geographic distribution of estimated admixture proportions were plotted using methods modified from Olivier François [118] using the MAPS, MAPTOOLS, and SPATIAL packages in R [119]–[122].

### African and non-African origin data partitions

After phasing the 260K dataset using the haplotypes inference algorithm implemented in version 2 of the SHAPEIT software [123], we partitioned the phased data from admixed HOA and MENA populations into African and non-African chromosome segments using the chromosome painting method implemented in the CHROMOPAINTER software [64]. This algorithm “paints” each target individual as a combination of segments from “donor” populations. As donors, we selected individuals from African and non-African populations without significant evidence for admixture: African populations used as donors were the Anuak, Ju/'hoansi, Mandenka, Mbuti, San, South Sudanese, and Yoruba; non-African ancestry populations used as donors were the Adygei, Basque, Bedouin, Brahui, Burusho, CEU, Druze, Gujarati (GIH), Hazara, Makrani, Orcadians, Pathan, Sardinians, and Saudi Arabians. For each admixed individual, each chromosome segment that was “painted” with 80% or greater confidence from African or non-African donor populations was assigned that origin. On average, 85% of each admixed individual's genome could be confidently partitioned. We then sampled from the painted segments to create 12 African ancestry and 12 non-African ancestry chromosomes for the admixed HOA population samples and the key neighboring admixed population samples of the Yemeni, Palestinians, Egyptians, and Mozabite (12 chromosomes was chosen as a compromise between maximizing sample size and maximizing the included populations). The Ari Blacksmith and Ari Cultivator samples were combined into a single Ari sample and the Ethiopian Somali and Somali samples were combined into a single Somali sample. The small original sample size of the Afar (n = 12) made it impossible to assemble enough African ancestry painted chromosome segments for this population and neither enough African nor non-African painted chromosome segments could be assembled for the Wolayta (original n = 8). To ensure that the African and non-African ancestry analyses would be directly comparable, we retained only those sites where 12 alleles could be selected from both the African and non-African painted segments across all populations; this reduced the starting 260K dataset to 4,340 SNPs (the “4K partitioned” dataset). Because we required a complete dataset with no missing data, the intersection across populations of available data considerably reduces the number of available sites (even though 85% of each individual genome could be confidently partitioned into African and non-African origin ancestries). Because of this, we had to use the 260K dataset, which unfortunately has reduced population representation, missing in particular most of the North African populations.

### Tests of gene flow and population structure in partitioned data

Using the 4K partitioned dataset, we evaluated the evidence for gene flow and population structure using Mantel tests, AMOVA, and population tree models. We tested for geographically mediated gene flow using Mantel tests of the correlation between genetic distance as measured by shared gene identity and geographic distance using the implementation in the R ADE4 package [124] with 10,000 random permutations of the data to estimate p-values. When appropriate, geographic distance was calculated both “as the crow flies” and through a northeastern African waypoint in Egypt. Population structure was assessed using AMOVA and population tree models. For AMOVA, we modeled structure at three levels, within populations, between populations within groups, and between groups, but focused on the tests for between group population structure. We used the AMOVA implementation in the R ADE4 package [124] with 10,000 random permutations of the data to estimate p-values. Population tree models were constructed following the method of Long and Kittles [67]. The fit of the data to the tree was assessed using their likelihood ratio statistic Λ. In most cases, the data deviate significantly from a perfect fit, which is not unexpected: Long and Kittles note that this statistic is likely to be very sensitive to any violation of the model assumptions. We assessed the improvement in fit from a less structured population tree to a more structured population tree using the K likelihood ratio statistic [67]. Both the Λ and K statistics are chi-squared distributed random variables.

### Ancestral population divergence

Population divergence times of the ADMIXTURE-inferred ancestral populations were estimated using the relationship 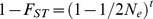 [73]. This estimate assumes that effective population sizes are known and have remained stable through time. We used a generation time of 30 years [125]–[127] and estimated minimum divergence times using an *N_e_* of 5,000, which is on the lower end of the *N_e_* values estimated for relevant HGDP populations [53]. Wright's original formulation of *F*_ST_ as a measure of differentiation resulting from the equilibrium between gene flow and genetic drift that is discussed in the main text is 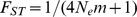 [73].

### Admixture tests and proportions

We formally tested for the presence of admixture in all study populations using the *f_3_*-statistic, the *D*-statistic, and a weighted LD statistic [62], [63]. Because a significant result for any one of these tests may be produced by histories other than admixture, we only report support for an admixture hypothesis when we found support for admixture from all three tests. To test for admixture between a sub-Saharan African and a non-African population, the *f_3_* test requires a reference population for each, which need not be the actual admixture source. For sub-Saharan Africa reference populations, we used populations that showed very little admixture of ancestral population components in the ADMIXTURE analysis: Mbuti Pygmies, Ju/'hoansi, HapMap3 Yoruba, South Sudanese, and Ari Blacksmith. For non-African reference populations, we used the HapMap3 CEU, Gujarati, and Tuscan populations in addition to Basque, Turkey, and Sardinian. The *f_3_* test was run for all other study populations for all possible pairs of reference populations. A strict Bonferroni correction was applied to control for multiple testing, only Z-scores less than −4 for the most negative *f_3_* statistic for each test population were considered significant. For those populations with significant *f_3_* statistics, the bounds of the admixture proportion were then estimated with the addition of a chimpanzee outgroup. The *f_3_* tests on the 90K and 260K datasets have more power, but return almost exactly the same *f_3_* statistic values (Table S7).

The test for admixture based on the *D*-statistic requires three populations in addition to the test population [62]. *D*-statistics significantly different from zero indicate either admixture or ancestral population structure. As in the *f_3_* test, the reference population suspected to be the source of admixture need not be the true source. We chose our population sets such that only positive values would reflect the admixture of interest. For sub-Saharan African and HOA test populations, the unrooted tree tested was ((African reference, test population), (Papuan, Basque)), where the African reference populations are the same as for the *f_3_* test. Since there is no indication in the literature of any African admixture in the Papuan population, any significantly positive *D*-statistic was taken as support for admixture between the test population and (a population related to) the Basque. For North African, Middle Eastern, and Eurasian test populations, the unrooted tree tested was ((Papuan, African reference), (Basque, test population)), where the African reference populations are the same as before. Again, since there is no indication in the literature of any admixture between Papuans and Basque, any significantly positive *D*-statistic indicates admixture between the test population and an African reference population. A strict Bonferroni correction was applied to control for multiple testing, only Z-scores greater than 4 for the most positive *D*-statistic for each test population were considered significant. The *D* tests on the 90K and 260K datasets have more power but recover indistinguishable *D* statistic values (Table S8).

Like the *f_3_* test, the weighted LD test in the ALDER software requires two reference populations, which need not be the actual admixture sources [63], and we used the same sets of non-African and sub-Saharan African reference populations. The test procedure implemented in ALDER controls for multiple testing across all the pairs of populations for each test population, but we still controlled for multiple testing across the whole family of tests using a strict Bonferroni correction, with only Z-scores greater than 3.2 considered statistically significant. The ALDER tests for admixture on the 90K and 260K datasets have more power but return similar results (Table S9).

We used three methods to calculate non-African admixture proportions in significantly admixed populations. First, we estimate the lower and upper bounds of non-African admixture using the bounding procedure allied with the *f_3_* admixture test [62]. This method requires an outgroup to the three populations in the *f_3_* test, but does not require a large sample, or even polymorphism, for the chosen outgroup. Therefore, following the recommendation in the description of this method, we used chimpanzee as the outgroup. Second, we estimated admixture proportions using the *f_4_* ratio estimation method [62]. The required number of populations and relationships among those populations for this method are as described for the *D* statistic test for admixture above, with the addition of an outgroup. Again, we used chimpanzee as the outgroup. Finally, for our third measure of non-African admixture proportions, we summed the proportions attributed to non-African ancestries from our ADMIXTURE analysis at K = 12.

### Admixture dating and simulations

We estimated the time of admixture for all populations identified as admixed using two LD-based methods: ROLLOFF [62], [70] and ALDER [63]. Following Pickrell et al. [17], we also compared the fit of single and double admixture models for admixed HOA populations. For comparison with other published admixture dates, we used the HapMap3 CEU and Yoruba populations as references. We also used the reference populations that gave the top *f_3_* statistic in the *f_3_* test for admixture and the reference populations giving the strongest signal in the ALDER test for admixture (sometimes these were the same). To verify the admixture date estimates calculated from the main (∼17K SNP) dataset are reliable, we ran ROLLOFF and ALDER on both the 90K and 260K datasets using the HapMap3 Yoruba and CEU as the reference populations. Using the main dataset, we estimate ROLLOFF admixture dates from 2.6–3.7 ka and ALDER admixture dates from 1.1–4.1 ka for admixed HOA population. The verification estimates are not meaningfully different from these, with ROLLOFF admixture dates from 2.6–3.7 ka and ALDER admixture dates from 1.2–3.3 ka for the 260K dataset (Table S10).

We simulated individuals of admixed ancestry following published protocols [70], [128]. We extracted 20 CEU and 40 Yoruba (YRI) individuals from a 260K SNP combined HapMap3 and HDGP dataset and phased them using fastPHASE [129]. These phased chromosomes were combined in episodic admixture scenarios, with two instances of admixture. We started with 20 CEU individuals and selected 20 random Yoruba individuals, and simulated admixture at time λ_0_ with admixture proportion α_0_ deriving from the Yoruba and 1 – α_0_ from the CEU. For each haploid admixed genome, we randomly selected one chromosome from each source population. We then created a vector of ancestry transition events along each chromosome by sampling with probability 1 – *e* ^−λ0*g*^, where *g* is the genetic distance in Morgans. Using this vector of transition event locations, we selected ancestry from the Yoruba chromosome with probability α_0_ at each transition. This procedure was repeated until we had 40 haploid admixed genomes. We then used these admixed chromosomes as a source population for the second episode of admixture at time λ_1_ with admixture proportion from α_1_ from the remaining 20 YRI individuals not selected for the first admixture. We randomly combined the 40 haploid admixed genomes into 20 diploid individuals. We chose to simulate 20 admixed individuals because the modal number of individuals in our admixed populations was about 20.

In our first set of simulations, we simulated admixture with λ_0_ equal to 50, 100, 150, or 200 generations and λ_1_ equal to 10 or 30 generations. Admixture proportion α_0_ was either 0.10 or 0.25 and admixture proportion α_1_ was 0.10. Three independent replicates were performed for each combination of parameters (48 simulations in total). The second set of simulations used λ_0_ equal to 50, 100, 150, 300, 500, 650, 850, 1000, or 1150 generations and λ_1_ equal to 30 generations. Admixture proportion α_0_ was 0.50 and admixture proportion α_1_ was 0.10. Again, three independent replicates were performed for each combination of parameters (27 simulations in total). Admixture dates were estimated for the simulation data using ROLLOFF and ALDER with the remaining unadmixed CEU and Yoruba individuals as the reference populations. In addition, we reduced the simulated data to the 16,766 SNPs present in the main dataset used to estimate admixture dates for the study populations and estimated admixture dates using ROLLOFF and ALDER for the same set of reference population pairs.

## Supporting Information

Figure S1 ADMIXTURE-inferred population average ancestry components for K = 2–20. The Ethiopian-specific Ethiopic ancestry component (dark purple) first appears at K = 11 and is stable from K = 12–20. The back-to-Africa Ethio-Somali ancestry component (green) first appears at K = 12 and is stable from K = 13–20.
(EPS)

Figure S2 Cross-validation error for K = 2–20 from the ADMIXTURE analysis. Ancestry proportions were estimated for K values ranging from 2 to 20, and cross-validation error was calculated for each value of K. The cross-validation error was minimized at K = 12.
(TIFF)

Figure S3 Tests for geographically mediated gene flow within and between HOA and MENA populations. The relationship between shared gene identity and inter-population distance is shown within and between HOA and MENA populations for both straight line distances and distance calculated using a waypoint through Egypt (where appropriate).
(EPS)

Figure S4 ROLLOFF and ALDER estimates of admixture times for simulated episodic admixture. We simulated two instances of admixture between CEU and YRI source populations using the 260K SNP dataset and estimated the time of admixture using ROLLOFF and ALDER. The 260K SNP dataset was also pruned down to ∼17K SNPs (to match the main dataset used in this study) and admixture dates were estimated from this reduced data as well. Loess lines were fit for each set of estimates: ROLLOFF 260K & 17K, ALDER 260K & 17K (thick solid lines). Overall, there is little difference in the estimates from the 260K and 17K simulated datasets. Previous simulation studies have characterized the ROLLOFF estimate of admixture time in episodic admixture scenarios as intermediate between the true dates of admixture; to better understand what this means, we plotted the weighted arithmetic, geometric, and harmonic means of the simulated admixture times (thin dotted lines). The ROLLOFF and ALDER admixture estimates are always heavily biased towards the more recent admixture. (A) The first admixture event was simulated between 50 and 200 generations ago, followed by a second admixture at 10 generations ago. During both episodes, 10% YRI ancestry was admixed. (B) Same as A, but with 25% YRI ancestry at the first (earlier) admixture. (C & D) Same as A and B, but with the second (more recent) admixture at 30 generations ago.
(EPS)

Figure S5 ROLLOFF and ALDER estimates for simulated ancient admixture followed by more recent admixture. same methods were followed as described for Figure S3, except a broader time range for the first (earlier) admixture was simulated, up to 1150 generations ago. In the first admixture, the YRI contributed 50% ancestry and in the second the YRI contributed 10% ancestry. Overall, there is little difference between the estimates based on the 260K SNP and the 17K SNP datasets. Both ROLLOFF and ALDER estimates are strongly biased towards the more recent admixture episode, with ALDER generally recovering values closer to the more recent admixture time.
(EPS)

Figure S6 Hierarchical population tree models for the non-African inferred ancestry components. Using the non-African IACs from the K = 12 ADMIXTURE analysis, a series of increasingly structured population tree models were constructed, starting from the unstructured tree (A), continuing through intermediately structured trees (B–D) to a final, fully bifurcating tree model (E). The fit to the data improves significantly with each more structured tree. While the final fully bifurcating is a significantly better fit to the data than any less structured tree, it deviates significantly from a perfect fit to the data.
(EPS)

Table S1 Population samples included in the main 17K, 90K, and 260K SNP datasets.
(XLSX)

Table S2 Results of f3 test for admixture, with estimated bounds of admixture for significantly admixed populations.
(XLSX)

Table S3 Results of D statistic test for admixture.
(XLSX)

Table S4 Results of ALDER test for admixture.
(XLSX)

Table S5 Non-African ancestry proportion estimated for significantly admixed study populations.
(XLSX)

Table S6 Ancestry proportions for each study population from ADMIXTURE ancestry components.
(XLSX)

Table S7 Verification of f3 test results from main (17K SNP) dataset in verification 90K and 260K datasets.
(XLSX)

Table S8 Verification of D statistic test results from main (17K SNP) dataset in verification 90K and 260K datasets.
(XLSX)

Table S9 Verification of ALDER test results from main (17K SNP) dataset in verification 90K and 260K datasets.
(XLSX)

Table S10 Verification of ROLLOFF and ALDER admixture date estimates from main (17K SNP) dataset in verification 90K and 260K datasets.
(XLSX)

Text S1 Affinities of the Ethio-Somali ancestry component. Three different analyses demonstrate that the Ethio-Somali ancestry component that is found at high frequencies in many HOA populations has a non-African origin.
(PDF)
