# Supplementary figures and images for: Early Back-to-Africa Migration into the Horn of Africa
Source: PLoS Genet. 2014 Jun 12;10(6):e1004393. doi: 10.1371/journal.pgen.1004393 (PMC4055572; doi:10.1371/journal.pgen.1004393)

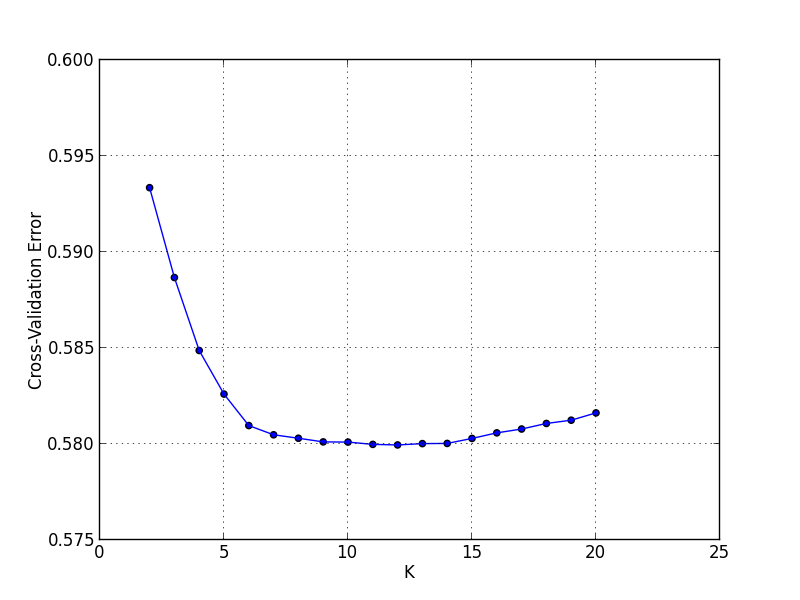

Supplement: Figure S2 — Cross-validation error for K = 2–20 from the ADMIXTURE analysis. Ancestry proportions were estimated for K values ranging from 2 to 20, and cross-validation error was calculated for each value of K. The cross-validation error was minimized at K = 12. (TIFF) [file pgen.1004393.s002.tif]
