# Supplementary material for: Early Back-to-Africa Migration into the Horn of Africa
Source: PLoS Genet. 2014 Jun 12;10(6):e1004393. doi: 10.1371/journal.pgen.1004393 (PMC4055572; doi:10.1371/journal.pgen.1004393)
Supplement: Text S1 — Affinities of the Ethio-Somali ancestry component. Three different analyses demonstrate that the Ethio-Somali ancestry component that is found at high frequencies in many HOA populations has a non-African origin. (PDF) [file pgen.1004393.s017.pdf]

***Supplementary Text S1: Affinities of the Ethio-Somali ancestry component***

Our analysis of the Ethio-Somali ancestry component revealed at K=12 produced three results that support its non-African affinities. First, the lowest pairwise $F_{ST}$ values estimated for the Ethio-Somali ancestral population from the ADMIXTURE analysis are with the Maghrebi and Arabian ancestral populations (0.074, 0.083) (the Maghrebi ancestry component derives from an early back-to-Africa migration into North Africa [1,2]). $F_{ST}$ values are on average lower between the Ethio-Somali component and non-African populations (mean=0.097) than with sub-Saharan African populations (mean=0.126) (see figure immediately below).

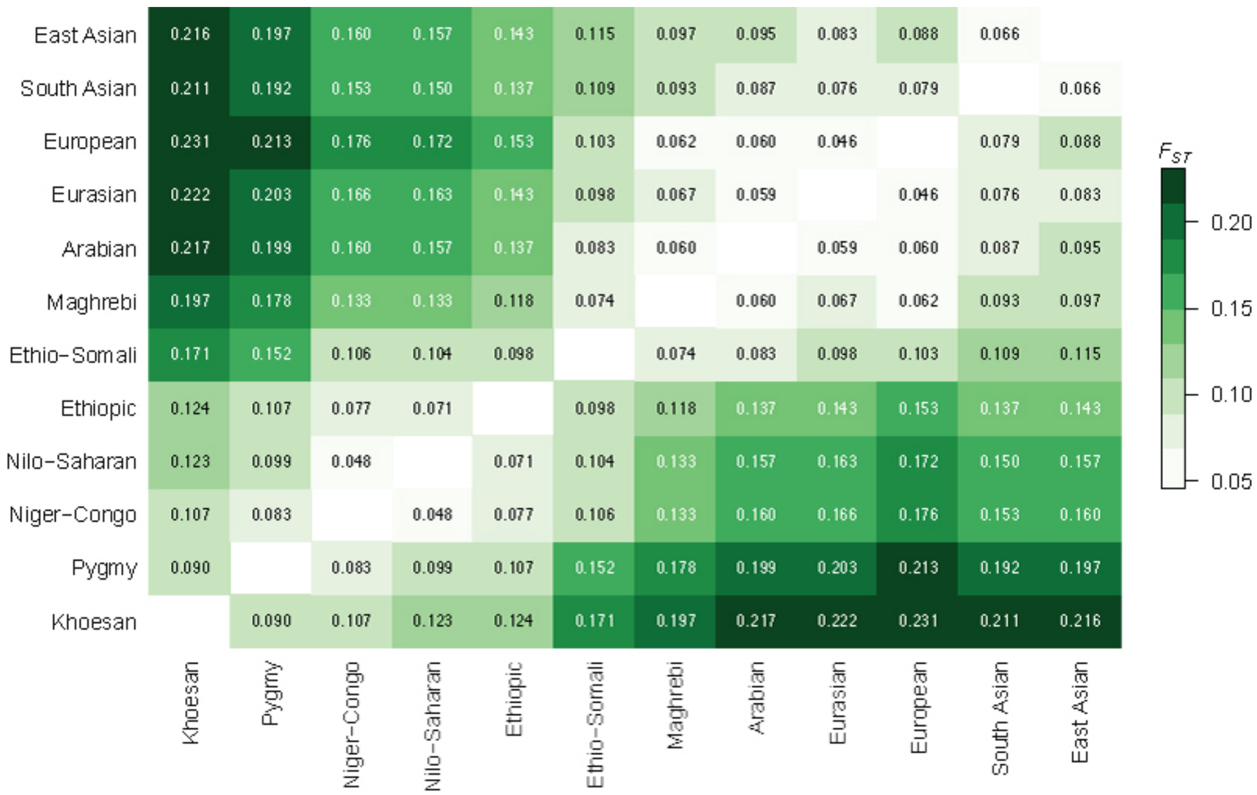


***Figure S1-1 – Pairwise* $F_{ST}$ *estimates show the non-African affinities of the Ethio-Somali.***

The Ethio-Somali ancestry component has the lowest pairwise $F_{ST}$ values with the Maghrebi and Arabian ancestral populations (0.074, 0.083). $F_{ST}$ values are on average lower between the Ethio-Somali component and non-African populations (mean=0.097) than with sub-Saharan African populations (mean=0.126).

Second, we conducted three formal tests for admixture and found that eight of the HOA populations had statistically significant signals of admixture with non-African populations for all three tests (Tables S2, S3, S4). For these eight populations, we estimated the proportion of non-African ancestry using both the admixture bounds extension of the $f_3$ test and $f_4$ ratio estimation [3]. We also summed the non-African ancestry components from the ADMIXTURE analysis with and without the Ethio-Somali component. The $f_3$ bounds and $f_4$ ratio estimates agree that all admixed HOA populations have around 50% non-African ancestry (see table immediately below). The ADMIXTURE estimates of non-African ancestry that do not include the Ethio-Somali values are substantially lower, averaging around 20%. Only if we include the Ethio-Somali component as non-African ancestry do the ADMIXTURE values become consistent with the $f_3$ bounds and $f_4$ ratio estimates.

***Estimates of non-African ancestry in admixed Horn of Africa populations.***

| **Population** | **$f_3$ bounds** | **$f_4$ ratio range** | **ADMIXTURE (-Ethio-Somali)** | **ADMIXTURE (+Ethio-Somali)** |
|---|---|---|---|---|
| Afar | 0.47 – 0.66 | 0.49 – 0.55 | 0.26 | 0.69 |
| Amhara | 0.48 – 0.61 | 0.52 – 0.71 | 0.30 | 0.65 |
| Ari Cultivator | 0.42 – 0.79 | 0.24 – 0.50 | 0.03 | 0.20 |
| Oromo | 0.58 – 0.76 | 0.42 – 0.65 | 0.24 | 0.55 |
| Ethiopian Somali | 0.38 – 0.60 | 0.43 – 0.65 | 0.11 | 0.64 |
| Somali | 0.39 – 0.59 | 0.43 – 0.65 | 0.09 | 0.66 |
| Tygray | 0.50 – 0.62 | 0.54 – 0.57 | 0.32 | 0.68 |
| Wolayta | 0.52 – 0.74 | 0.39 – 0.60 | 0.17 | 0.44 |

Third, the first dimension of the IBS MDS analysis appears to represent the proportion of non-African ancestry (Main Text Figure 1). If so, there should be a positive relationship between non-African ancestry proportions estimated from the ADMIXTURE analysis (K=12) and the MDS first dimension values. We fit linear models testing this relationship for all ancestry components that contribute at least 5% ancestry to two or more HOA populations (see table immediately below). As predicted, there is a positive relationship between the first MDS dimension and Arabian or Eurasian ancestry (and a negative relationship between the first MDS dimension and Nilo-Saharan or Ethiopic ancestry). The relationship between Ethio-Somali ancestry and the first MDS dimension is both statistically significant and positive ($p = 1.3 \times 10^{-6}$, $R^2 = 0.14$).

***Relationship between ADMIXTURE ancestry and first MDS dimension[1].***

| | **Direction** | **$R^2$** | **p** |
|---|---|---|---|
| Nilo-Saharan | negative | 0.67 | $< 2.2 \times 10^{-16}$ |
| Ethiopic | negative | 0.10 | $2.0 \times 10^{-6}$ |
| Ethio-Somali | positive | 0.14 | $1.3 \times 10^{-6}$ |
| Arabian | positive | 0.52 | $1.3 \times 10^{-15}$ |
| Eurasian | positive | 0.02 | n.s. |

[1] For those ancestry components present at 5% or greater in two or more Horn of Africa populations.

Taken in combination, these three lines of evidence offer strong support to the hypothesis that the Ethio-Somali ancestry component has a non-African origin, and results are consistent with earlier findings of substantial non-African ancestry in HOA populations [4–6].

***Supplemental Text References***


1. Henn BM, Botigué LR, Gravel S, Wang W, Brisbin A, et al. (2012) Genomic Ancestry of North Africans Supports Back-to-Africa Migrations. PLoS Genet 8: e1002397. doi:10.1371/journal.pgen.1002397.

2. Sánchez-Quinto F, Botigué LR, Civit S, Arenas C, Ávila-Arcos MC, et al. (2012) North African Populations Carry the Signature of Admixture with Neandertals. PLoS ONE 7: e47765. doi:10.1371/journal.pone.0047765.

3. Patterson NJ, Moorjani P, Luo Y, Mallick S, Rohland N, et al. (2012) Ancient Admixture in Human History. Genetics 192: 1065–1093. doi:10.1534/genetics.112.145037.

4. Cavalli-Sforza LLL, Menozzi P, Piazza A (1994) History And Geography Of Human Genes. Princeton: Princeton University Press. 1114 p.

5. Passarino G, Semino O, Quintana-Murci L, Excoffier L, Hammer MF, et al. (1998) Different genetic components in the Ethiopian population, identified by mtDNA and Y-chromosome polymorphisms. Am J Hum Genet 62: 420–434. doi:10.1086/301702.

6. Pagani L, Kivisild T, Tarekegn A, Ekong R, Plaster C, et al. (2012) Ethiopian Genetic Diversity Reveals Linguistic Stratification and Complex Influences on the Ethiopian Gene Pool. Am J Hum Genet 91: 83–96. doi:10.1016/j.ajhg.2012.05.015.
